# Supplementary material for: Neuronal network dysfunction in a model for Kleefstra syndrome mediated by enhanced NMDAR signaling
Source: Nat Commun. 2019 Oct 30;10:4928. doi: 10.1038/s41467-019-12947-3 (PMC6821803; doi:10.1038/s41467-019-12947-3)
Supplement: Supplementary file 1 — Supplementary Information [file 41467_2019_12947_MOESM1_ESM.pdf]

Supplementary Information

Neuronal network dysfunction in a model for Kleefstra syndrome mediated by enhanced NMDAR signaling

Monica Frega et al.

Supplementary Figures

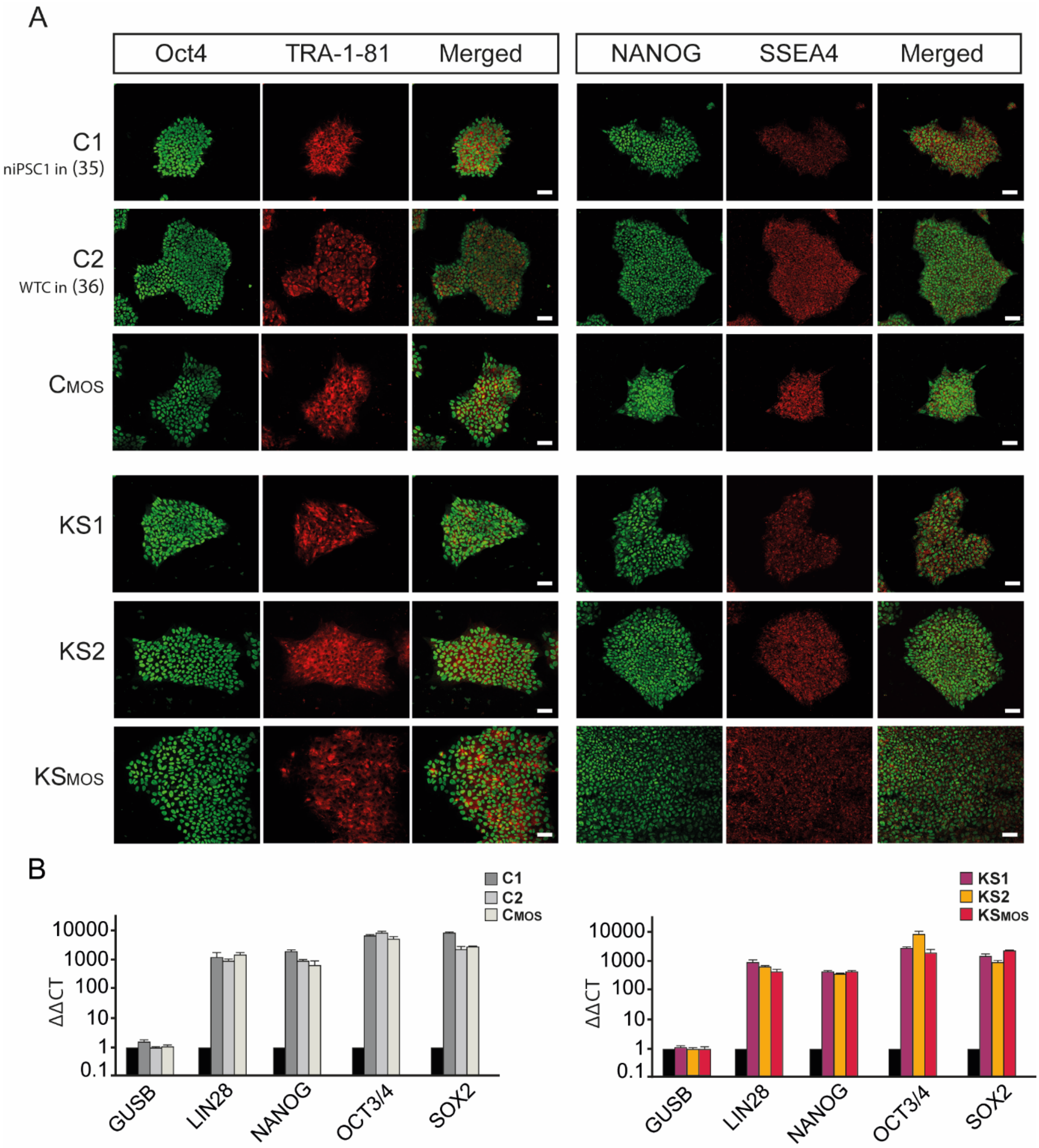

Supplementary Fig. 1. Characterization of control and KS patients iPS cells. A) Representative images of iPS cell colonies from 3 control and 3 KS lines (C_1, C_2, C_{MOS}; KS_1, KS_2, KS_{MOS}) stained for different pluripotency marker (scale bar 50 μM). All lines used in this study were examined for the expression of the nuclear marker OCT4 and NANOG (green) and the surface marker TRA-1-81 and SSEA4 (red) by means of immunocytochemistry. **B)** QPCR data for control (left panel, C_1, C_2 and C_{MOS}) and KS patient (right panel, KS_1, KS_2 and KS_{MOS}) derived iPSCs showing an upregulation of pluripotency markers in iPS cells relative to their expression in corresponding parent fibroblasts (black).

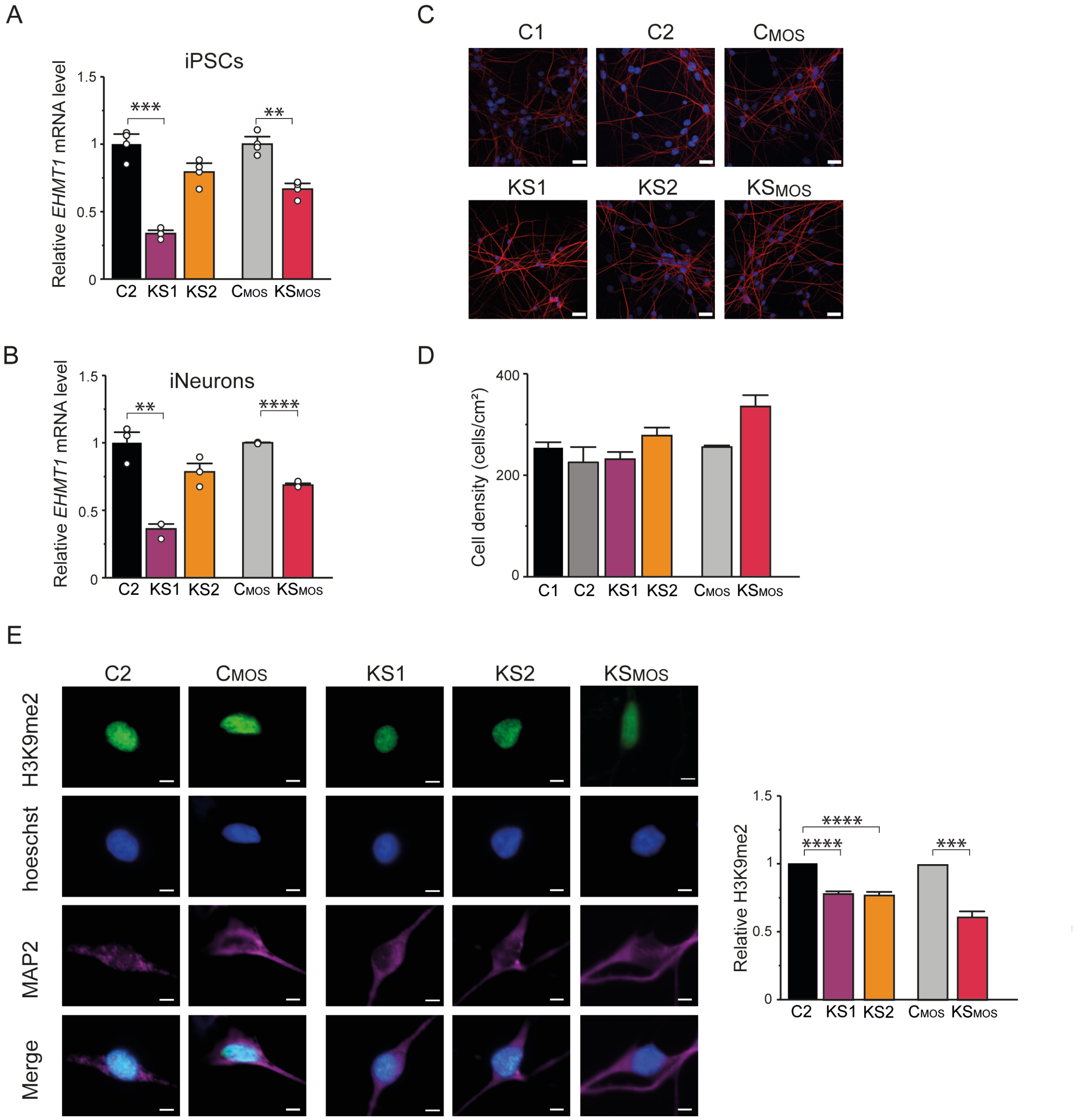

Supplementary Fig. 2. Characterization of control and KS iPS cells and iNeurons. A-B) Graphs respectively showing relative *EHMT1* mRNA level in **A)** iPSCs and in **B)** iNeurons. Each dot represents one experiment: n=3 for all lines (i.e. controls: C_2, C_{MOS}; KS: KS_1, KS_2, KS_{MOS}). Values from KS_1 and KS_2 are normalized to C_2; values from KS_{MOS} are normalized to C_{MOS}. **C)** Representative figure of control and KS iNeurons stained for MAP2 at DIV 21 (scale bar 50 µM). **D)** Quantification of the density of neurons derived from 3 controls and 3 KS iNeurons at 21 DIV. **E)** Representative figure of control and KS iNeurons stained for H3K9me2 (green) and MAP2 (pink) at DIV 3 (scale bar 5 µm) and quantification of H3K9me2 mean fluorescence intensity (MFI). n=38 for C_2; n=15 for C_{MOS} ; n=130 for KS_1; n=52 for KS_2; n=24 for KS_{MOS}. Values from KS_1 and KS_2 are normalized to C_2; values from KS_{MOS} are normalized to C_{MOS}. Data represent means ± SEM. ** $P<0.005$, *** $P<0.0005$, one-way ANOVA test and post hoc Bonferroni correction was performed between controls and KS-derived cultures.

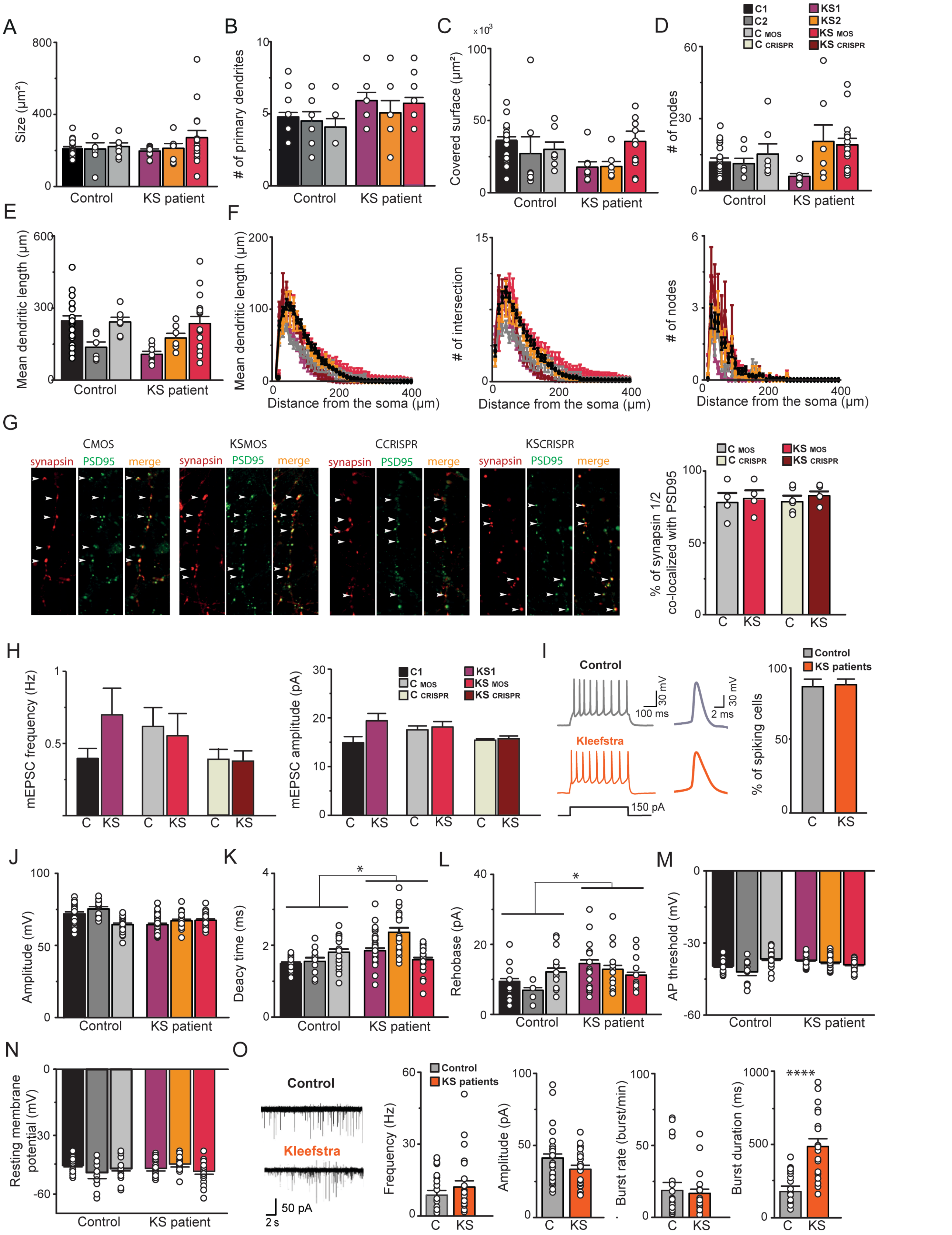

Supplementary Fig. 3. Single-cell characterization of control and KS iNeurons. **A-E)** Graphs respectively showing the **A)** neuronal size, **B)** number of primary dendrites, **C)** covered surface, **D)** number of nodes and **E)** mean dendritic length for neurons derived from 3 control and 3 KS iNeurons. **F)** Graph showing the Sholl analysis on mean dendritic length, number of intersections and number of nodes. Dendrite intersections were counted at 10 µm intervals. Each dot represents one cell: n=22 for C_1; n=7 for C_2; n=7 for C_{MOS}; n=7 for KS_1; n=8 for KS_2; n=16 for KS_{MOS}. **G)** Representative figure of neurons derived from C_{MOS}, KS_{MOS}, C_{CRISPR} and KS_{CRISPR} stained for synapsin 1/2 (red) and PSD95 (green) at

DIV 21 (scale bar 10 μm) to indicate the formation of functional synapses with a pre- and post-synaptic site and quantification of the co-localization (n=4 for C_{MOS}, n=4 for KS_{MOS}, n=6 for CC_{RISPR} and n=5 for KS_{CRISPR}). **H)** Graph showing the frequency and amplitude of mEPSCs received by control (C_1, C_{MOS}, C_{CRISPR}) and KS patient (KS_1, KS_{MOS}, KS_{CRISPR}) derived neurons. n=10 for C_1, n=11 for C_{MOS}, n=9 for KS_1, n=12 for KS_{MOS}, n=8 for C_{CRISPR} , n=8 for KS_{CRISPR}. **I)** Representative example traces of action potentials generated by control and KS patient iNeurons (grey and orange respectively) and graph showing the percentage of cells generating a train of 10 action potentials (n=60 for C; n=85 for KS). **J-M)** Graphs respectively showing **J)** the amplitude of the action potentials (AP), **K)** the decay time, **L)** the rheobase (i.e. current needed to generate an AP), **M)** the AP threshold and **N)** the resting membrane potential for neurons derived from 3 control and 3 KS iNeurons. Each dot represents one cell: n=24 for C_1; n=12 for C_2; n=24 for C_{MOS}; n=33 for KS_1; n=22 for KS_2; n=30 for KS_{MOS}. **O)** Example traces and quantification of whole cell voltage-clamp recordings of spontaneous excitatory postsynaptic currents (sEPSCs) in control (C_1, C_{MOS}, C_{CRISPR}) and KS iNeurons (KS_1, KS_{MOS}, KS_{CRISPR}). Each dot represents one cell: n=21 for control; n=21 for KS**.** Data represent means ± SEM. * $P<0.05$, **** $P<0.0001$. One-way ANOVA test and post hoc Bonferroni correction was performed between controls and KS patient-derived cultures.

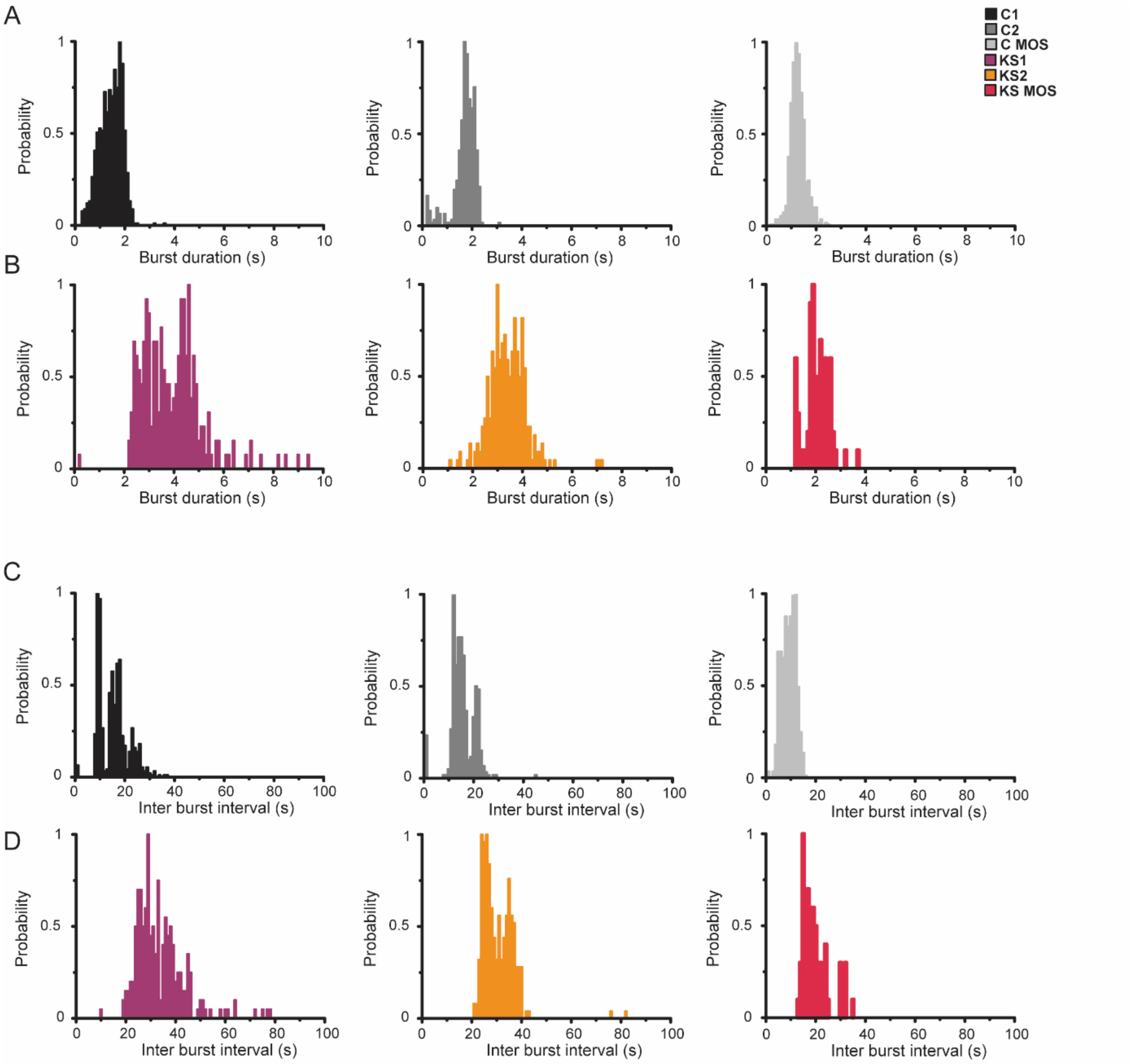

Supplementary Fig. 4. Distribution of network burst duration and interval for control and KS neuronal networks. **A-B)** Graphs showing the network burst duration distribution for **A)** control- and **B)** Kleefstra patient-derived neuronal networks (bin size=100 ms) **C-D)** Graphs showing the network burst interval distribution for **C)** control- and **D)** Kleefstra patient-derived neuronal networks (bin size=1 s).

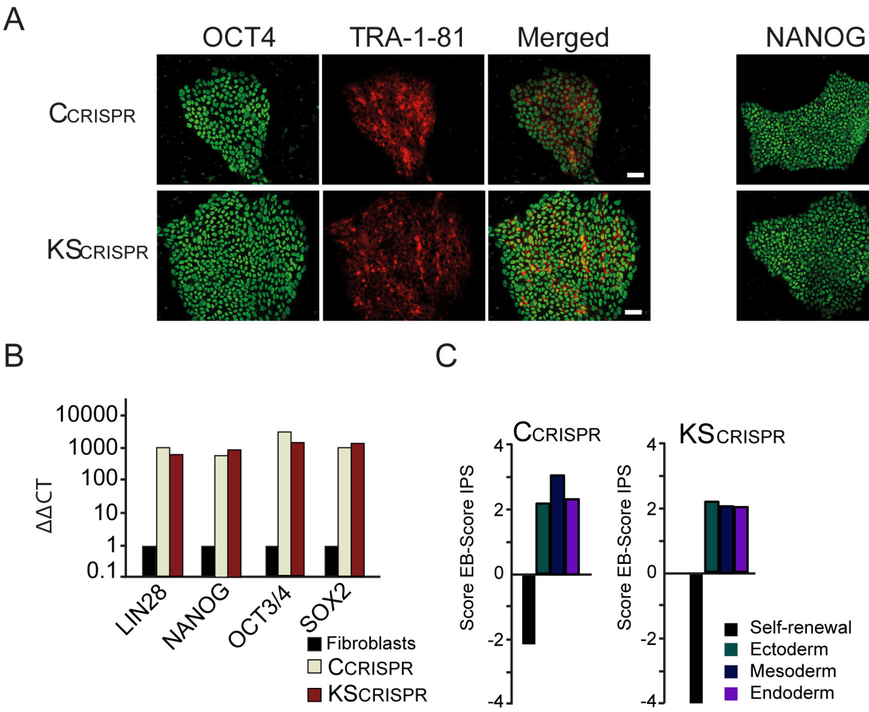

D

	CCRISPR	KSCRISPR
	allele1/allele2	allele1/allele2
C___1563023_10	VIC/FAM	VIC/FAM
C___1801627_20	VIC/VIC	VIC/VIC
C___2728408_10	FAM/FAM	FAM/FAM
C___1250735_20	FAM/FAM	FAM/FAM
C___15935210_10	VIC/VIC	VIC/VIC
C___7431888_10	VIC/FAM	VIC/FAM
C___3227711_10	FAM/FAM	FAM/FAM
C___1902433_10	VIC/FAM	VIC/FAM
C___30044763_10	VIC/FAM	VIC/FAM
C___31386842_10	VIC/FAM	VIC/FAM
C___33211212_10	VIC/FAM	VIC/FAM
C___26524789_10	FAM/FAM	FAM/FAM
C___11821218_10	FAM/FAM	FAM/FAM
C____43852_10	VIC/FAM	VIC/FAM
C___1670459_10	FAM/FAM	FAM/FAM
C___8924366_10	VIC/FAM	VIC/FAM
C___1007630_10	VIC/FAM	VIC/FAM
C___11522992_10	VIC/FAM	VIC/FAM
C___7421900_10	VIC/VIC	VIC/VIC
C___10076371_10	VIC/FAM	VIC/FAM
C___26546714_10	VIC/FAM	VIC/FAM
C___1122315_10	VIC/VIC	VIC/VIC
C___27402849_10	VIC/VIC	VIC/VIC
C___7457509_10	VIC/FAM	VIC/FAM
C___29619553_10	VIC/FAM	VIC/FAM
C___11710129_10	VIC/VIC	VIC/VIC
C___2953330_10	FAM/FAM	FAM/FAM
C___1027548_20	VIC/FAM	VIC/FAM
C___8850710_10	VIC/VIC	VIC/VIC
C___1083232_10	VIC/VIC	VIC/VIC
C___16205730_10	VIC/FAM	NOAMP
C___8938211_20	FAM/FAM	FAM/FAM

E

Guide ID	Chr	Strand	Position	Sequence	# mismatches	Score	Ontarget	Gene	Primers
gRNA1									
17578567	chr2	1	15905213	CCTTACAGGCAGTTCCGGGGAGG	3	1,127119	False	None	FWD: AAGTAGCTGCCCTTCTCTGGCCTTGG<br>REV: TCTGGAGATGCTAAGTGAAGATCTGAGGCG
17578567	chr13	-1	25607933	AATAGCAGGCAGTTCCAGCGGAG	4	0,620617	False	None	FWD: AAGCTCCGCCTCCTGGGTTCACACCATTC<br>REV: ATCACCTGAGGCCAGGAGTTCGAGACTAGC
17578567	chr10	1	56810492	AATAACTGGCAGTTCCTGCGTAG	4	0,423881	False	None	FWD: CAAAGCTGTATTGGCATATCCTC<br>REV: GGCATGAAATTCTAGTGATGTAACC
17578567	chr11	-1	8375789	TCTGGGAGGCAGTTCCGGCCGGG	4	0,406681	False	None	FWD: CCAGCTGCTGCTCCTCAGGG<br>REV: ACTGCAAGGTGAGCCGCCAA
gRNA3									
17578569	chr2	1	15905215	TTACAGGCAGTTCCGGGGAGGGG	2	5,225	False	None	FWD: AAGTAGCTGCCCTTCTCTGGCCTTGG<br>REV: TCTGGAGATGCTAAGTGAAGATCTGAGGCG
17578569	chr12	-1	51236842	TAGCCGCCGGTTCCGGCGAGGGG	4	0,561634	False	NM_182559	FWD: CACGAACAGGTACTGCCCTCTGCATG<br>REV: TAACCGACCTAACAGGATTCTTGCTGGAC
17578569	chr11	-1	44883535	GAACAGGCCTTCCCGGCGAGTGG	4	0,409264	False	None	FWD: TGGCACATGATAGGCACTCAACAGCC<br>REV: CCATGCCTCTGATGGATCTGAGAGTGGTAC
17578569	chr11	-1	869112	GGACAGGCATTTCCAGCGAGGGG	4	0,383985	False	NM_023947	FWD: GCCTGGCAGTGATAGAGCTGAGAGCTCCAC<br>REV: TCCAGGCGGAGGCTTTCTCAAGTGTGG

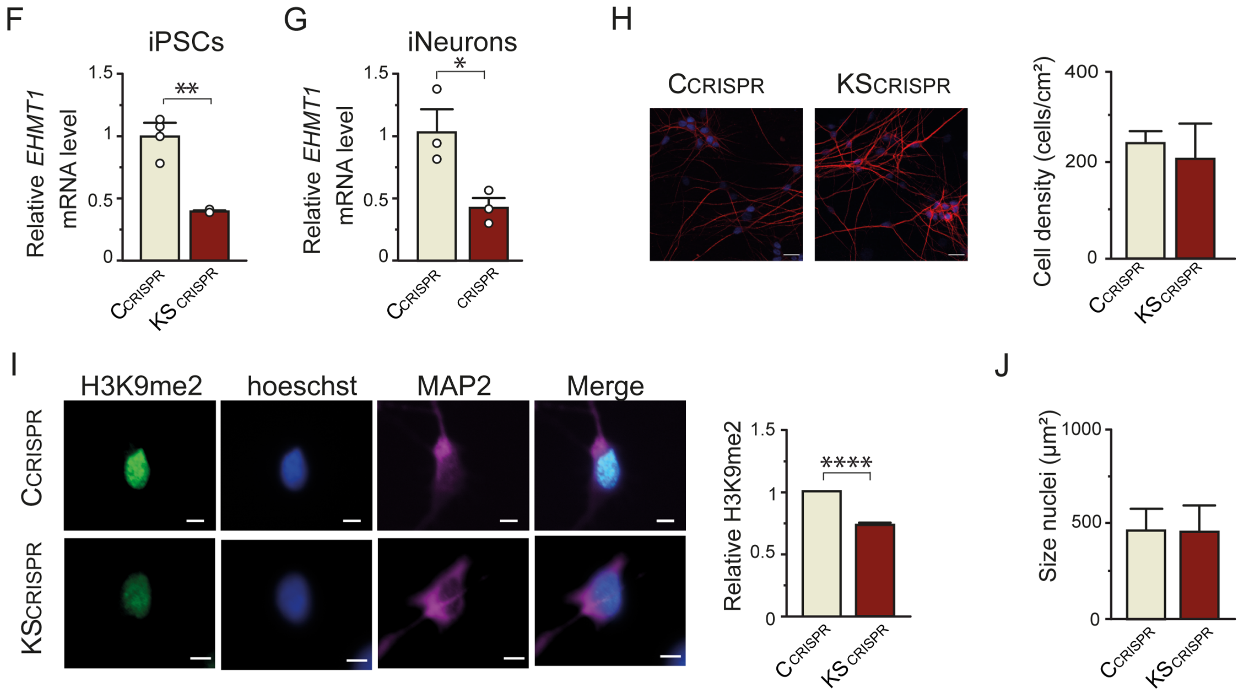

Supplementary Fig. 5. Characterization of CRIPR/cas9-edited iPS cell and iNeurons. A) Representative images of iPS cell colonies (C_{CRISPR} and KS_{CRISPR}) stained for different pluripotency marker (scale bar 50 µM). C_{CRISPR} and KS_{CRISPR} iPS cells lines were examined for the expression of the nuclear marker OCT4 and NANOG (green) and the surface marker TRA-1-81 and SSEA4 (red) by means of immunocytochemistry. **B)** QPCR data for control (C_{CRISPR}) and CRISPR edited (KS_{CRISPR}) line showing an upregulation of pluripotency markers in iPSCs relative to their expression in the fibroblasts. **C)** Quantitative analysis of tri-lineage differentiation potential; both C_{CRISPR} and KS_{CRISPR} lines have the capacity to differentiate towards all three germ layers **D)** DNA fingerprinting: the provided genome-edited iPSC line shows identical SNP profile with the corresponding parent iPS cell line used for gene targeting. **E)** Top four off-target sites of each gRNA have been sequenced and no mutations were detected. **F-G)** Graphs respectively showing relative *EHMT1* mRNA level in **F)** iPS cells and **G)** iNeurons. Each dot represents one experiment: n=3 for C_{CRISPR} and KS_{CRISPR}. Values from KS_{CRISPR} are normalized to C_{CRISPR}. **H)** Representative figure of C_{CRISPR}- and KS_{CRISPR}-derived neuronal networks stained for MAP2 at DIV 21 (scale bar 50 µm) and quantification of the density C_{CRISPR} and KS_{CRISPR} iNeuons at DIV 21. **I)** Representative figure of control and KS iNeurons stained for H3K9me2 (green) and MAP2 (pink) at DIV 3 (scale bar 5 µm) and quantification of H3K9me2 mean fluorescence intensity (MFI) in C_{CRISPR} and KS_{CRISPR} iNeurons. n=36 for C_{CRISPR}; n=140 for KS_{CRISPR}. Values from KS_{CRISPR} are normalized to C_{CRISPR}. **J)** Quantification of the nuclei size. n=61 for C_{CRISPR} and n=63 for KS_{CRISPR}. Data represent means ± SEM. ** $P<0.005$, *** $P<0.0005$, one-way ANOVA test and post hoc Bonferroni correction was performed between controls and KS-derived cultures.

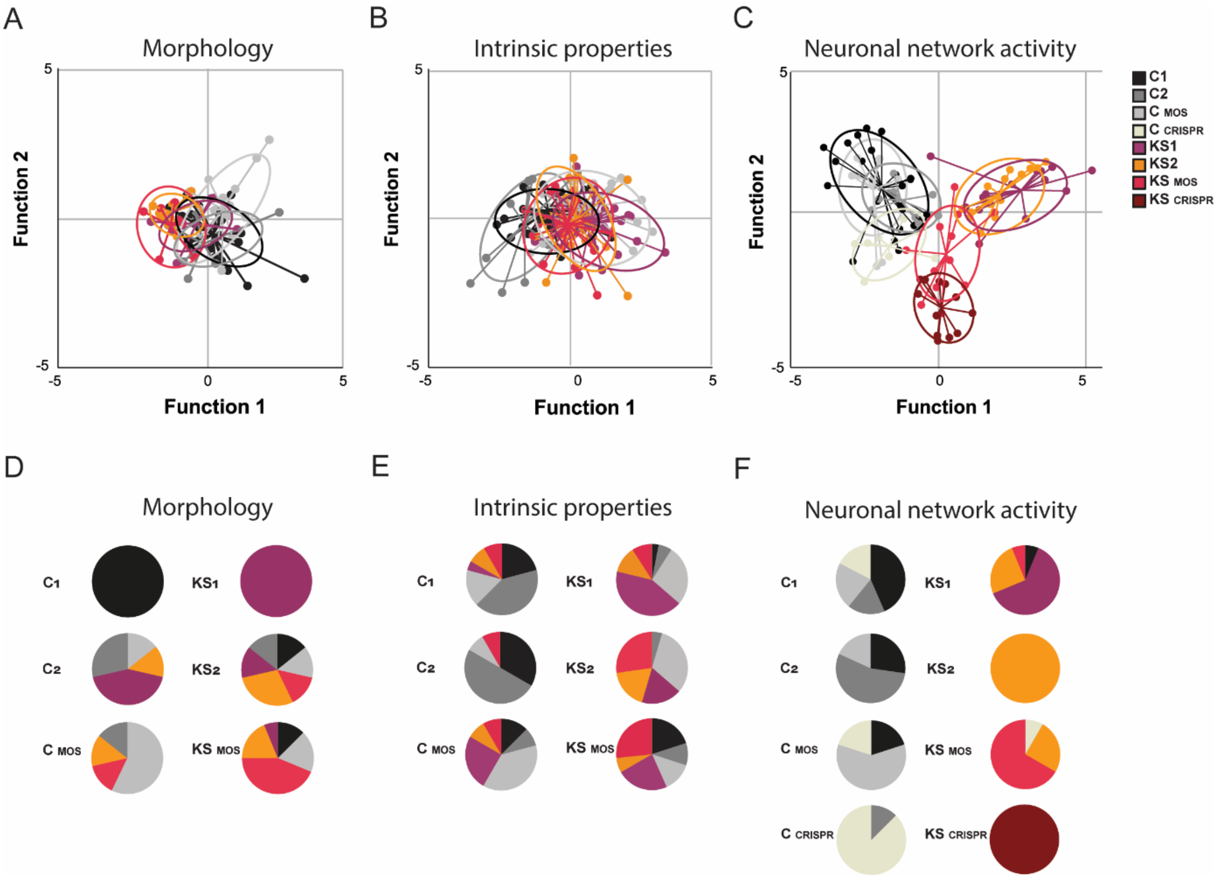

Supplementary Fig. 6. Discriminant analysis classification of control and KS iNeurons. A-C) Discriminant function analysis with canonical discriminant functions classifies C_1, C_2, C_{MOS}, KS_1, KS_2 and KS_{MOS} lines based on **A)** the neuronal size, the number of primary dendrites, the covered surface, the mean dendritic length and the number of nodes (i.e. morphology) (43% correct classification), **B)** the amplitude of the AP, the decay time, the rheobase and the AP threshold (i.e. intrinsic properties) (32% correct classification) and **C)** discriminant function analysis with canonical discriminant functions classifies C_1, C_2, C_{MOS}, C_{CRISPR}, KS_1, KS_2, KS_{MOS} and KS_{CRISPR} lines based on the firing rate, network bursting rate, network burst duration, percentage of spike outside network burst and coefficient of variability of the inter-burst interval (i.e. neuronal network activity) (70% correct classification). Group envelopes (ellipses) are centered on the group centroids. The experiment for each line and the ellipses are shown in different colors (i.e. black, dark grey, light grey and white for C_1, C_2, C_{MOS}, C_{CRISPR} and purple, yellow, light red and dark red for KS_1, KS_2, KS_{MOS}, KS_{CRISPR} respectively). Morphology: each dot represents one cell: n=22 for C_1; n=7 for C_2; n=7 for C_{MOS}; n=7 for KS_1; n=8 for KS_2; n=16 for KS_{MOS}. Intrinsic properties: each dot represents one cell: n=24 for C_1; n=12 for C_2; n=24 for C_{MOS}; n=33 for KS_1; n=22 for KS_2; n=30 for KS_{MOS}. Neuronal network activity: Each dot represents one experiment: n=23 for C_1; n=10 for C_2; n=10 for C_{MOS}; n=7 for C_{CRISPR}; n=15 for KS_1; n=15 for KS_2; n=12 for KS_{MOS} and n=12 for KS_{CRISPR}. **D-F)** Pie diagrams indicating the predicted group membership for each line based on **D)** neuronal morphology, **E)** intrinsic properties and **F)** neuronal network activity.

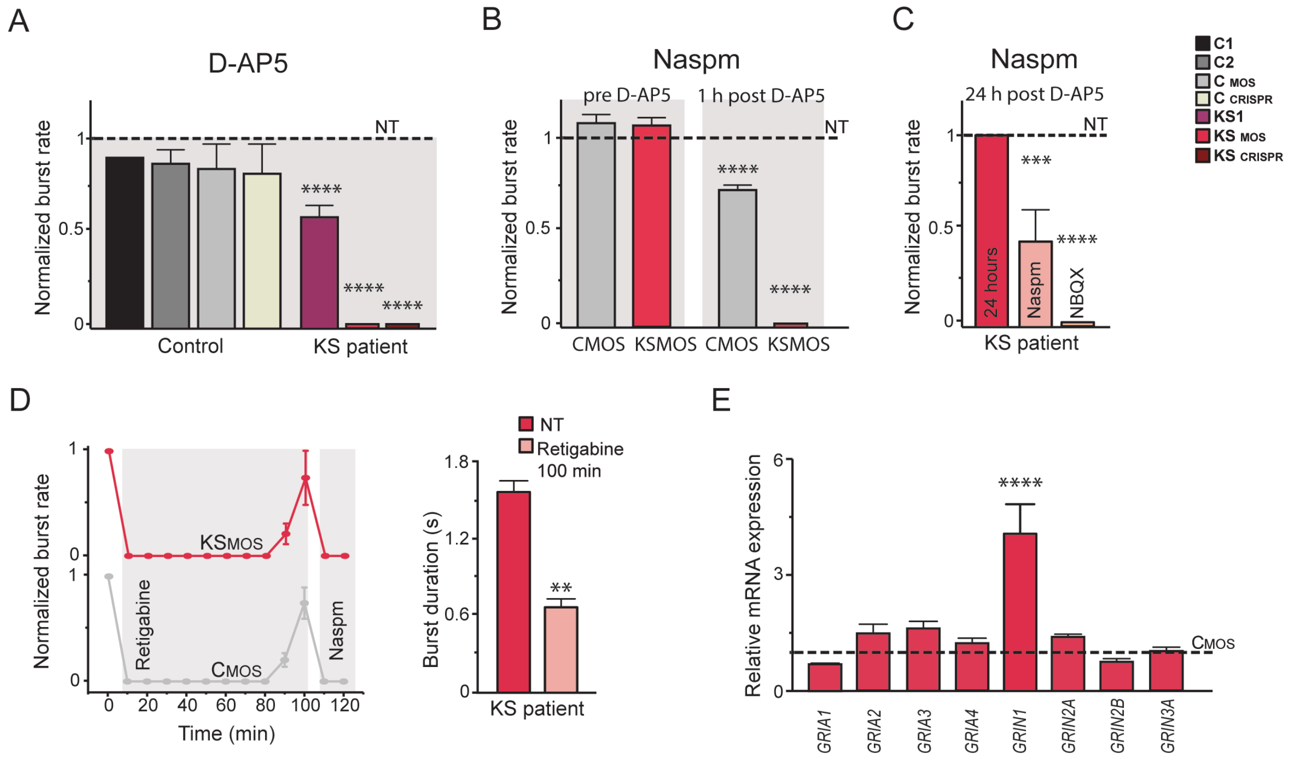

Supplementary Fig. 7. Effect of AMPA- and NMDA-blockage on control and KS network. A) Graph showing the effect of D-AP5 (60 µM) treatment on the neuronal network burst frequency for control- and KS networks at DIV 28. The D-AP5 response (10 min after application) is shown for C_1, C_2, C_{MOS}, C_{CRISPR}, KS_1, KS_{MOS} and KS_{CRISPR}. The values are normalized by the non-treated (NT) condition. The values are shown in different colors (i.e. black, dark grey, light grey for C_1, C_2, C_{MOS} and purple, light red and dark red for KS_1, KS_{MOS}, KS_{CRISPR} respectively). n=6 for C_1, n=6 for C_2, n=3 for C_{MOS}, n=10 for KS_1, n=14 for KS_{MOS}, n=14 for KS_{CRISPR}. **B)** Graph showing the effect of Naspm (10 µM) treatment on the neuronal network burst frequency for C_{MOS} and KS_{MOS} in two conditions: non-treated cultures; after 1 hour of treatment with D-AP5. The values are normalized to the non-treated (NT) condition. The values are shown in different colors (i.e. light grey for C_{MOS} and light red for KS_{MOS} respectively). n=6 for C_{MOS} and KS_{MOS} not treated; n=10 for C_{MOS} and KS_{MOS} D-APV treated. **C)** Graph showing the effect of Naspm (10 µM) on KS_{MOS} neuronal network bursting activity 24 hours after D-AP5 treatment (NT indicates network burst activity before D-AP5 treatment). Naspm alone is not completely blocking the network bursting activity anymore. The network bursting activity is blocked when NBQX (50 µM) is added. n=3. The values are normalized to the bursting frequency 24 hours after D-AP5 treatment. **D)** Retigabine (10 µM) effect on C_{MOS} and KS_{MOS} neuronal network activity during time. After 100 min, Naspm (10 µM) was added in the medium and the network burst disappeared in both C_{MOS} and KS_{MOS}. The values are normalized to the non-treated (NT) condition. Graph showing the duration of the network burst of KS_{MOS} before and after 100 min of Retigabine (10 µM) treatment. n=3. **E)** Bar graph showing relative *GRIA1*, *GRIA2*, *GRIA3*, *GRIA4*, *GRIN1*, *GRIN2A*, *GRIN2B* and *GRIN3A* mRNA expression in control and KS iNeurons at DIV 28. KS_{MOS} mRNA expression is normalized to C_{MOS} (n=3). Data represent means ± SEM. * $P<0.05$, *** $P<0.0005$, **** $P<0.0001$, one-way ANOVA test and post hoc Bonferroni correction was performed between controls and KS networks and Mann-Whitney test was performed between two groups.

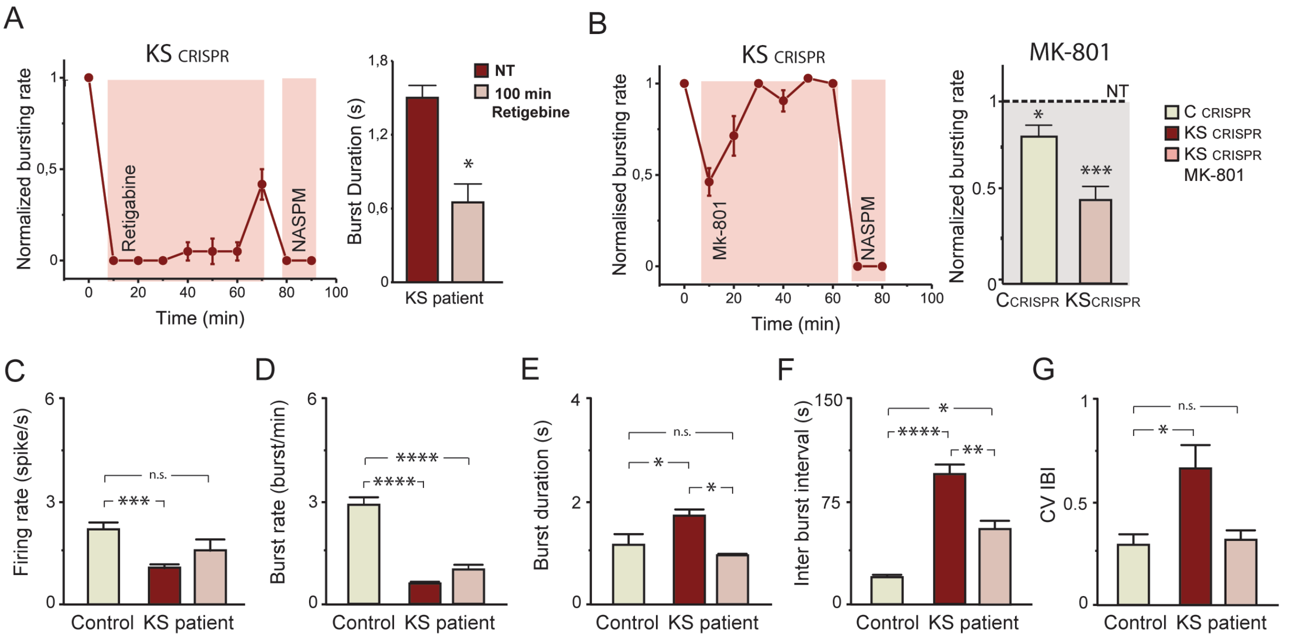

Supplementary Fig. 8. NMDAR antagonist MK-801 rescues KS$_{CRISPR}$ network phenotypes. A) Retigabine (10 µM) effect on KS$_{CRISPR}$ neuronal network activity during time. After 70 min, Naspm (10 µM) was added in the medium and the network burst disappeared. The values are normalized to the non-treated (NT) condition. Graph showing the duration of the network burst of KS$_{CRISPR}$ before and after 100 min of Retigabine (10 µM) treatment. n=4. **B)** MK-801 (1 µM) effect on KS$_{CRISPR}$ neuronal network activity (DIV 28). After 60 min, Naspm was added. Graph showing the effect of MK-801 (1 µM) treatment on the neuronal network burst frequency for C$_{CRISPR}$ and KS$_{CRISPR}$ derived neuronal network 20 min after application. The values are normalized to the non-treated (NT) condition. **C-G)** Quantification of network properties as indicated, n=8 for C$_{CRISPR}$; n=6 for KS$_{CRISPR}$ and n=6 for KS$_{CRISPR}$ treated with MK-801. Data represent means ± SEM. * $P<0.05$, ** $P<0.005$, *** $P<0.0005$, **** $P<0.0001$, one-way ANOVA test and post hoc Bonferroni correction was performed between conditions.

Supplementary Tables

EHMT1	Forward	GCTGGGAGAAGAGACACCTA
	Reverse	TGCTGGCATCGCTGTTT
GRIA1	Forward	GCAGCAGTGGAAGAATAGTGATG
	Reverse	ATCACCTTCACCCCATCGTA
GRIA2	Forward	GCTTGGTGCTAAATTGCTGT
	Reverse	TCCAAGAAAAGTAGAGCATCCA
GRIA3	Forward	TTCCCACTGGAGGCATGTG
	Reverse	CATCAGCAATATTCGTGTCATGC
GRIA4	Forward	TGCTGCAACTAAGACCTTCGTTAC
	Reverse	TCGAGTATCCCCTGTCTGTGTC
GRIN1	Forward	CGCCGCTAACCATAAACAAC
	Reverse	GGGGAATCTCCTTCTTGACC
GRIN2A	Forward	AGCTGCTACGGGCAGATG
	Reverse	CCTGGTAGCCTTCCTCAGTG
GRIN2B	Forward	GGAGTTCTGGTTCCTACTGGG
	Reverse	TCTCATGGGAACAGGAATGG
GRIN3A	Forward	CATTCCAGTGATCAGCATCG
	Reverse	GGGTAAGGAGGAGGAAGTCG
PPIA (housekeeping)	Forward	CATGTTTTCCTTGTTCCCTCC
	Reverse	CAACACTCTTAACTCAAACGAGGA

Supplementary Table 1. Primers designed for RT-qPCR experiments targeting human transcripts.

***BDNF*_pr4**	Forward	TGT TTG GTC GGC TAG AAA GC
	Reverse	CCCAGATAACGTTACACCAAG
***GRIN1*_pr2**	Forward	ACCCCAAGATCGTCAACATT
	Reverse	GGCATTGAGCTGAATCTTCC
***PPIA*_pr1**	Forward	TTGGCCTGACCTAATGTTGG
	Reverse	TCCTATGGCTTCCCTTTCAG

Supplementary Table 2. Primers designed for ChIP-qPCR experiments targeting human promoter regions.
