# Supplementary material for: Neuronal network dysfunction in a model for Kleefstra syndrome mediated by enhanced NMDAR signaling
Source: Nat Commun. 2019 Oct 30;10:4928. doi: 10.1038/s41467-019-12947-3 (PMC6821803; doi:10.1038/s41467-019-12947-3)
Supplement: Supplementary file 3 — Description of Additional Supplementary Files [file 41467_2019_12947_MOESM3_ESM.pdf]

Description of Additional Supplementary Files

File Name: Supplementary Data 1
Description: Overview of all statistics. For each experiment the statistical test and exact P value is given.
