# Supplementary material for: Neuronal network dysfunction in a model for Kleefstra syndrome mediated by enhanced NMDAR signaling
Source: Nat Commun. 2019 Oct 30;10:4928. doi: 10.1038/s41467-019-12947-3 (PMC6821803; doi:10.1038/s41467-019-12947-3)
Supplement: Supplementary file 5 — Reporting Summary [file 41467_2019_12947_MOESM5_ESM.pdf]

nature research

Corresponding author(s): Nael Nadif Kasri

Last updated by author(s): Aug 6, 2019

Reporting Summary

Nature Research wishes to improve the reproducibility of the work that we publish. This form provides structure for consistency and transparency in reporting. For further information on Nature Research policies, see Authors & Referees and the Editorial Policy Checklist.

Statistics

For all statistical analyses, confirm that the following items are present in the figure legend, table legend, main text, or Methods section.

n/a	Confirmed	
☐	☒	The exact sample size (*n*) for each experimental group/condition, given as a discrete number and unit of measurement
☐	☒	A statement on whether measurements were taken from distinct samples or whether the same sample was measured repeatedly
☐	☒	The statistical test(s) used AND whether they are one- or two-sided<br>*Only common tests should be described solely by name; describe more complex techniques in the Methods section.*
☐	☒	A description of all covariates tested
☐	☒	A description of any assumptions or corrections, such as tests of normality and adjustment for multiple comparisons
☐	☒	A full description of the statistical parameters including central tendency (e.g. means) or other basic estimates (e.g. regression coefficient) AND variation (e.g. standard deviation) or associated estimates of uncertainty (e.g. confidence intervals)
☒	☐	For null hypothesis testing, the test statistic (e.g. *F*, *t*, *r*) with confidence intervals, effect sizes, degrees of freedom and *P* value noted<br>*Give P values as exact values whenever suitable.*
☒	☐	For Bayesian analysis, information on the choice of priors and Markov chain Monte Carlo settings
☒	☐	For hierarchical and complex designs, identification of the appropriate level for tests and full reporting of outcomes
☒	☐	Estimates of effect sizes (e.g. Cohen's *d*, Pearson's *r*), indicating how they were calculated

Our web collection on statistics for biologists contains articles on many of the points above.

Software and code

Policy information about availability of computer code

Data collection	MEA data was collected using Multiwell Screen (Multichannel systems, Germany).<br>Single cell activity was recorded using Signal (CED, Cambridge, UK)
Data analysis	Neuronal morphology recostruction was performed used Neurolucida 360 software (MBF–Bioscience, Williston, ND, USA).<br>MEA recordings were analysed in Multiwell-Analyzer (Multichannel systems, Germany) and Matlab R2018b using a custom softwares packages.<br>Intrinsic electrophysiological properties were analysed using Signal and MatLab (MathWorks, MA, USA), while mEPSCs were analysed using MiniAnalysis 6.0.2 (Synaptosoft Inc, Decatur, GA, USA) .<br>Acute slice electrophysiological data was analyzed in Clampfit 10.7 (Molecular Devices).<br>Immunocytochemestry signals were quantified using FIJI software and ImageJ analysis tools.<br>The statistical analysis for all experiments was performed using GraphPad Prism 5 (GraphPad Software, Inc., CA, USA).<br>All detailed statistical analysis can be found in Table S3

For manuscripts utilizing custom algorithms or software that are central to the research but not yet described in published literature, software must be made available to editors/reviewers. We strongly encourage code deposition in a community repository (e.g. GitHub). See the Nature Research guidelines for submitting code & software for further information.

Data

Policy information about availability of data

All manuscripts must include a data availability statement. This statement should provide the following information, where applicable:

- Accession codes, unique identifiers, or web links for publicly available datasets
- A list of figures that have associated raw data
- A description of any restrictions on data availability

n.a.

Field-specific reporting

Please select the one below that is the best fit for your research. If you are not sure, read the appropriate sections before making your selection.

☒ Life sciences ☐ Behavioural & social sciences ☐ Ecological, evolutionary & environmental sciences

For a reference copy of the document with all sections, see nature.com/documents/nr-reporting-summary-flat.pdf

Life sciences study design

All studies must disclose on these points even when the disclosure is negative.

Sample size	Sample size for slice physiology experiments and cell cultures were determined based on previously published literature and experience in the lab.
Data exclusions	Data was excluded if values were more then 3 standard deviations distanced from the mean. Furthermore, neuronal networks were excluded from the data set if a clear degradation of cell culture health was visible over development, which was assessed by light microscopy
Replication	Reproducibility of MEA and single cell data, immunocytochemistry, neuronal recontruction, RT-qPCR, Chip-qPCR was addressed by keeping culturing and recording conditions, as well as timing of the experiments constant between batches (i.e. one batch is one differentiation). Of each iPSCs line, multiple batches were recorded subsequently.
Randomization	Wt and heterozygous mice used for electrophysiology experiments were litter-matched, genotypes were blind to the experimenter, no randomisation was performed.<br>Human iPSCs derived neuronal networks on MEA were all analysed using the same analysis settings and after that grouped based on genotype. This approach was also used for single cell electrophysiology.<br>ICC images were analysed blind and then grouped per genotype. This approach was used for neuronal morphological reconstruction, RT-qPCR and Chip-qPCR too.
Blinding	All analysis were done blinded to the genotype of the recorded cells.<br>Activity on MEA were all analysed using the same analysis settings which therefore controls for bias. This approach was also used for single cell electrophysiology.<br>ICC images were randomised manually and counted blinded by an unbiased author.

Reporting for specific materials, systems and methods

We require information from authors about some types of materials, experimental systems and methods used in many studies. Here, indicate whether each material, system or method listed is relevant to your study. If you are not sure if a list item applies to your research, read the appropriate section before selecting a response.

Materials & experimental systems

n/a	Involved in the study	
☐	☒	Antibodies
☒	☐	Eukaryotic cell lines
☒	☐	Palaeontology
☐	☒	Animals and other organisms
☒	☐	Human research participants
☒	☐	Clinical data

Methods

n/a	Involved in the study	
☒	☐	ChIP-seq
☒	☐	Flow cytometry
☒	☐	MRI-based neuroimaging

Antibodies

Antibodies used	Used primary antibodies: mouse anti-MAP2 (1:1000; Sigma M4403); guinea pig anti-MAP2 (1:1000; Synaptic Systems 188004); guinea pig anti-synapsin 1/2 (1:1000; Synaptic Systems 106004); mouse anti-PSD95 (1:50; Thermo Fisher Scientific MA1-045); rabbit anti-GFAP (1:500; Abcam ab7260), mouse anti-pan axon (1:1000; Covance SMI-312R), rabbit anti-H3K9me2 antibody (1:500, Millipore 07-441); mouse anti-NMDAR1 (1:1000; ThermoFisher Scientific 54.1).<br>Secondary antibodies: goat anti-guinea pig Alexa Fluor 647 (1:2000, Invitrogen A-21450); goat anti-rabbit Alexa Fluor 488 (1:2000, Invitrogen A-11034); goat anti-rabbit Alexa Fluor 568 (1:2000, Invitrogen A-11036); goat anti-mouse Alexa Fluor 488 (1:2000, Invitrogen A-11029); goat anti-mouse Alexa Fluor 568 (1:2000, Invitrogen A-11031).
Validation	Antibodies were validated on the used species by using a dilution series of the primary antibody based on manufacturers recommendations. Also a secondary antibody control was included during the validation.

Animals and other organisms

Policy information about studies involving animals; ARRIVE guidelines recommended for reporting animal research

Laboratory animals	Slice physiology was performed on C57BL/6 of both genders at postnatal days 21 and 24
Wild animals	n.a.
Field-collected samples	n.a.
Ethics oversight	Animal experiments were conducted in conformity with the Animal Care Committee of the Radboud University Nijmegen Medical Centre, The Netherlands, and conform to the guidelines of the Dutch Council for Animal Care and the European Communities Council Directive 2010/63/EU.

Note that full information on the approval of the study protocol must also be provided in the manuscript.
